# Carcinoid Heart Disease on Computed Tomography

**DOI:** 10.5334/jbsr.2201

**Published:** 2020-09-11

**Authors:** Jean-Philippe Hardy, Benoît Ghaye

**Affiliations:** 1 Cliniques universitaires Saint Luc, BE

**Keywords:** carcinoid heart disease, carcinoid syndrome, thorax, oncology, carcinoid tumor, valve, scanner

## Abstract

**Teaching Point:** Carcinoid tumors can release hormones responsible of cardiac valves fibrosis known as carcinoid heart disease.

## Case

**Figure 1:**
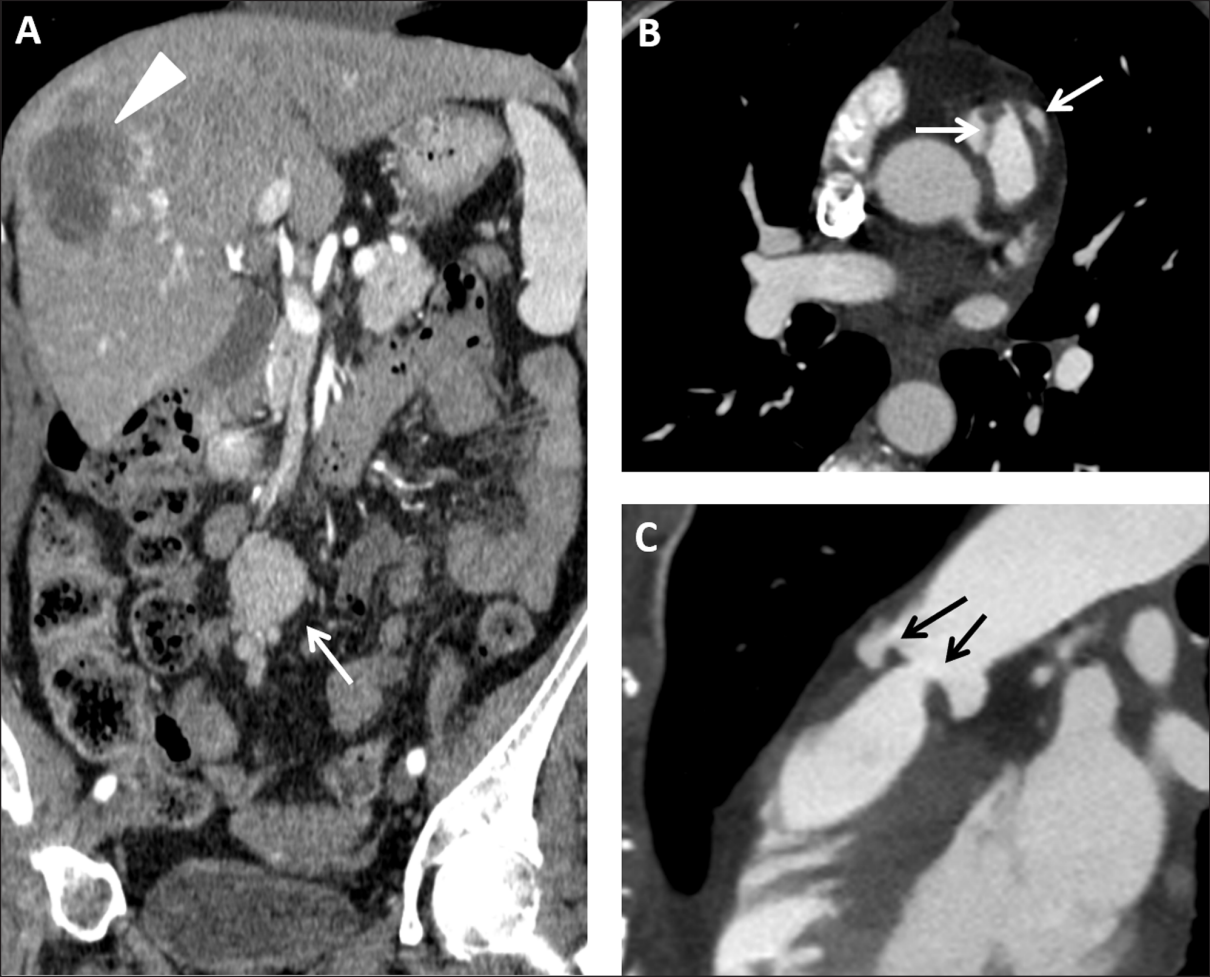


**Figure 2:**
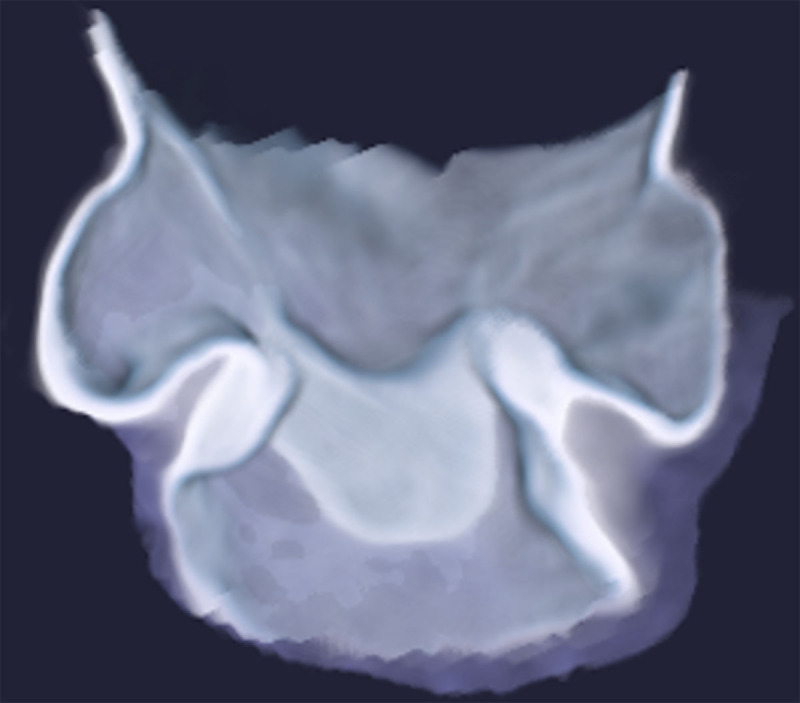


A 68-year-old-woman presented with dyspnea (blood oxygen level: 90%), abdominal pain, diarrhea, and a flush. Body CT showed a strongly enhancing mesenteric mass (Figure 1A, arrow) and multiple hepatic nodules showing peripheral contrast enhancement (Figure 1A, arrowhead). At the thoracic level, the right cardiac valves showed a nodular thickening of their leaflets. The thickened pulmonary valve is illustrated on transversal (Figure 1B, arrows), sagittal (Figure 1C, arrows), and volume-rendered (Figure 2) views. Liver biopsy revealed metastasis from small bowel neuroendocrine tumor. Clinical findings were therefore related to a typical carcinoid syndrome induced by metastatic carcinoid tumor. In this context, the thickening of the pulmonary valve corresponds to carcinoid heart disease. This diagnosis was confirmed by echocardiography which showed moderate pulmonary valve insufficiency and severe tricuspid insufficiency. Our patient underwent surgical valvular replacement.

## Comments

Carcinoid tumors are uncommon (1.9 per 100,000 persons per year worldwide). Forty-two percent of gastrointestinal carcinoids tumors are localized in the small intestine. Carcinoid syndrome concerns less than 10% of neuroendocrine tumors and is supposed to be induced by various vasoactive hormones released by the tumor, including serotonin. These agents are metabolized or inactivated by the liver, lungs, and brain. Therefore, carcinoid syndrome from a gastrointestinal tumor usually cannot take place without metastasis that bypasses the normal liver degradation. Carcinoid syndrome is characterized by diarrhea, abdominal pain, cutaneous flush, bronchospasms, and cardiac valvular disease. Approximately 50% of patients with carcinoid syndrome will develop carcinoid heart disease consisting in valvular fibrosis and leading to stenosis and/or insufficiency. The most frequent involvement of the right-sided cardiac valves (90%) is likely explained by lung inactivation of hormones produced by the tumor. The tricuspid valve is the most frequently affected valve. Pathophysiology of fibrosis remains discussed although hormones such as serotonin seem to be involved.

Transthoracic echocardiography is the first-line imaging method, although technically limited for right cardiac valves due to their retrosternal location. CT provides valuable anatomical information of the valves and main pulmonary artery, providing reproducible data helping surgical planning and diagnosis. However, CT functional valvular assessment is limited due to limited temporal resolution. Carcinoid heart disease may be suspected at CT based on morphological features. Indeed, whereas normal cardiac valve appears thin and non-nodular on CT, valves affected by carcinoid heart disease appear thickened [1]. In the context of metastatic carcinoid tumor, thickening of a right-sided cardiac valve on CT should therefore rise the suspicion of carcinoid heart disease. The only specific treatment in severe cases is surgical valvular replacement.

## References

[1] Farhood S, Atul G, S Yen H. CT and MR imaging of the Pulmonary valve. Radiology. 2014; 34: 51–71. DOI: 10.1148/rg.341135026 24428282

